# Posttraumatic stress symptoms after solid-organ transplantation: preoperative risk factors and the impact on health-related quality of life and life satisfaction

**DOI:** 10.1186/1477-7525-11-111

**Published:** 2013-07-04

**Authors:** Andreas Baranyi, Till Krauseneck, Hans-Bernd Rothenhäusler

**Affiliations:** 1 Department of Psychiatry, University of Medicine of Graz, Auenbruggerplatz 31, Graz 8036, Austria; 2 Department of Psychiatry, Ludwig-Maximilians University of Munich, Nußbaumstraße 7, Munich 80336, Germany

**Keywords:** Solid-organ transplantation, Intensive care unit, Posttraumatic stress symptoms, Preoperative risk factors, Health-related quality of life, Life satisfaction

## Abstract

**Background:**

Solid-organ transplantations (SOT) are usually life-saving high-tech medical procedures. The transplantation itself and the intensive care unit stay could be traumatic stressors triggering posttraumatic stress symptoms (PTSS). Our retrospective follow-up study aimed to explore preoperative risk factors of PTSS in a cohort of SOT recipients, and we investigated how PTSS are associated with health-related quality of life (HRQOL) and life satisfaction.

**Methods:**

126 SOT recipients were enrolled in this investigation. Psychiatric examination of all SOT candidates based on the Transplant Evaluation Rating Scale was carried out before SOT, and after SOT, recipients completed the PTSS-10, the SF-36 and the FLZ.

**Results:**

After the surgical intervention 19 (15.1%) SOT recipients had clinical significant PTSS. Preoperative risk factors for developing postoperative PTSS were: 1.) preexisting psychiatric morbidity, 2.) history of retransplantation, 3.) chronic benzodiazepine consumption, 4.) age, and 5.) type of transplantation.

SOT-related PTSS were associated with maximal decrements in HRQOL and life satisfaction. The following HRQOL and life satisfaction domains were affected: Physical Functioning, Role Physical, Pain, General Health, Vitality, Social Functioning, Role Emotional, Mental Health, Occupation/Work and Character/Own Skills.

**Conclusion:**

SOT recipients may face a major risk of transplantation- and treatment-related PTSS and the development of impairments to HRQOL and life satisfaction.

## Background

Great advances have been made in the field of transplantation surgery, and the success of solid-organ transplantations (SOTs) is no longer measured solely by their effect on physical morbidity and mortality but also by their impact on transplant recipients’ mental health and wellbeing [1-12].

It needs to be mentioned that for those directly affected, SOTs are usually life-saving but nevertheless stressful and serious interventions associated with the risk of numerous medical complications (e.g. acute rejection, infections, bleeding). It is therefore not surprising that psychiatric morbidity (e.g. delirium, affective disorders, anxiety disorders and adjustment disorders) is a frequent finding in patients after SOT. The available frequency data for psychiatric morbidity after liver transplantation range from 54% 3 months after transplantation to 22% in a 3.8 years follow-up. Observed rates for anxiety disorders (e.g. panic disorder, generalized anxiety disorder, and posttraumatic stress disorder related to the transplant experiences) in transplant populations range from 3% to 33% in the first years after transplantation [13-16]. The pretransplant evaluation- and waiting period result in stress and uncertainty in SOT-candidates [17,18]. Furthermore, the transplantation itself and the intensive care unit (ICU) stay might be traumatic stressors that can trigger posttraumatic stress symptoms (PTSS) [1].

Health-related quality of life (HRQOL) and life satisfaction are now an important measure of outcome in SOT recipients [18-22]. Collis et al. [23] and Rothenhäusler et al. [24] demonstrated that there is a significant association between impaired HRQOL and psychiatric morbidity in patients after liver transplantation.

### Research questions

Knowledge about possible predictors and risk factors of posttraumatic stress symptomatology after SOT is still very limited. Only few studies have aimed to explore preoperative risk factors of PTSS after transplantation. We therefore examined variables such as type of transplantation, history of retransplantation, preexisting psychiatric morbidity, preoperative chronic benzodiazepine consumption, preoperative alcohol abuse, and important sociodemographic characteristics. Furthermore, we investigated the association of PTSS, HRQOL and life satisfaction.

The trauma criterion was homogeneously defined as SOT followed by ICU treatment.

## Method

### Participants and procedure

During the study period a total of 280 patients underwent SOT at the Transplantation Center of the Klinikum Grosshadern, Ludwig-Maximilians-University, Munich, Germany.

Before SOT, the transplantation candidates were evaluated by experienced consultation-liaison psychiatrists using the observer-rating Transplant Evaluation Rating Scale (TERS) [25]. 65 of these 280 patients died before study inclusion. 215 eligible SOT recipients were contacted in a letter and were asked to complete a research battery, which included an author-compiled clinical questionnaire, the PTSS-10 [26,27], the Medical Outcome Study Form (SF-36) [28] and the life satisfaction questionnaire “Fragebogen zur Lebenszufriedenheit” (FLZ) [29]. 126 transplantation recipients returned the questionnaires, at a mean of 24.9 months (SD=11.9) after transplantation. 84 patients responded to our letter but refused to join the study and 5 patients were untraceable. Respondents with complete data were similar on the sociodemographic and treatment parameters to those lost due to refusal or untraceability.

Independent of type of transplantation the data analysis was performed for the whole sample according to the homogeneously definition of the trauma criterion as SOT followed by ICU treatment. The SF-36 data were compared with data from normative population whose individuals were drawn with respect to age and gender in a pairwise matching from a large data base (n=3000) used for the validation of the SF-36 in Germany.

The study was approved by the Institutional Review Board of the Ludwig-Maximilians-University of Munich. Data protection met the standards set by German law.

### Psychiatric history

The psychiatric history is based on a psychiatric clinical interview before transplantation performed by experienced consultation-liaison psychiatrists (H.-B. R., T. K.).

### Questionnaires

#### Transplant Evaluation Rating Scale (TERS)

The TERS scale classifies the level of adjustment in psychosocial functioning among SOT candidates and covers the different dimensions of psychosocial functions (preexisting psychiatric morbidity [e.g. PTSS, PTSD], substance abuse, compliance, coping strategies, cognitive performance) [25].

#### Sociodemographic and transplantation characteristics questionnaire

After SOT, an author-compiled questionnaire was used to obtain information about the sociodemographic and transplantation characteristics. Demographic variables included age, gender, years of education and/or vocational training, employment and marital status at the time of psychiatric assessment. The patients’ employment status was categorized as paid work (full- or part-time) or no paid work (disability, retired, unemployed). Marital status was categorized as married, single, divorced, or widowed. Further, a preexisting psychiatric morbidity before SOT, pre- and postoperative chronic benzodiazepine consumption and alcohol abuse were recorded.

Collected transplantation characteristics were the indications for heart transplantation (ischemic or dilated cardiomyopathy), orthotopic liver transplantation (alcoholic liver disease, infectious hepatitis, primary biliary cirrhosis, malignancy or other), and lung transplantation (emphysema and chronic obstructive pulmonary disease, pulmonary fibrosis, pulmonary hypertension or other). Postoperative medical complications, acute rejections and a history of retransplantation were observed.

#### Psychometric tests

**PTSS 10**[26,27]: The PTSS-10 is a self-rating 10-item scale based on the Diagnostic and Statistical Manual of Mental Disorders (DSM-IV-TR), that measures the presence and intensity of posttraumatic stress symptoms. The German version of the PTSS-10 (range: 10–70 points) has been successfully validated in patients with PTSD after prolonged ICU treatment and posttransplant, and has been proved to be a reliable scale. The questionnaire shows a high internal consistency (Cronbach’s alpha=0.93), and a high test-retest reliability (intraclass correlation coefficient alpha=0.89). The criterion validity was demonstrated by ROC curve analyses resulting in a sensitivity of 77.0% and a specificity of 97.5%. The cut-off point for clinically significant PTSS is 35 points [26,27].

**SF-36**[28]: To assess HRQOL, we applied the psychometrically well-validated German translation of the Medical Outcome Study Form SF-36, a 36-item self-rating questionnaire that covers eight health-related domains. The domains are: Physical Functioning, Role Physical, Pain, General Health, Vitality, Social Functioning, Role Emotional, and Mental Health.

Each domain yields a score ranging from 0 to 100 (best). In the vast majority of the published studies, the internal-consistency data of the SF-36 exceed 0.8 [28].

**“Fragebogen zur Lebenszufriedenheit” (FLZ)**[29]: The FLZ is a widely used and psychometrically well-validated self-rating life satisfaction questionnaire that covers ten life satisfaction domains: health, occupation/work, income/financial security, leisure time/hobbies, partner relationship, family life/children, character/own skills, sexuality, friends/acquaintances and housing/living conditions. Each of the ten domains covers seven items that are rated on a seven-point scale (ranging from “very unsatisfied” to “very satisfied”). In addition to gathering data on domain-specific life satisfaction, the FLZ also allows for an evaluation of general life satisfaction. This is calculated as the sum total of seven of ten domains (the domains occupation/work, partner relationship and family life/children are not included). The internal-consistency data of the FLZ are between 0.82 and 0.95 [29].

### Statistical analyses

Descriptive statistics were produced based on demographic, treatment-related and psychometric data (PTSS-10, SF-36, FLZ) and are presented as mean and standard deviation (SD). We applied the non-parametric χ^2^ - test, the Fisher’s exact test and the Mann–Whitney-U test to test differences between patients with or without PTSS and the non-parametric Wilcoxon signed-rank test to compare solid-organ transplantation recipients with healthy controls matched by age and gender. All statistic tests were two-tailed, with significance set at p<0.05. In case of multiple comparisons (SF-36, FLZ), an alpha adjustment (Bonferroni) was used. By means of multivariate analyses in the form of multiple regression models, the level of influence by the identified risk factors and PTSS-associated manifestation in the SF-36 and FLZ dimensions in regard to a PTSS symptomatology was examined.

All statistical analyses were performed with IBM SPSS Statistics 20.0 for Windows.

## Results

### Sociodemographic characteristics, clinical characteristics and preoperative psychiatric morbidity

All 126 participants (69.0% men, 31.0% women) were Caucasian, and the mean age was 52.4 (SD=11.6) years.

Preoperative psychiatric diagnoses based on the TERS and an examination by experienced consultation-liaison psychiatrists were recorded more frequently in patients with liver transplantation compared with patients after heart or lung transplantation (χ^2^ =53.657; df=34; p=0.017). Main preoperative psychiatric diagnoses in the subgroup of patients with liver transplantation were substance abuse (n=9), personality disorder [narcissistic or histrionic] (n=6), and adjustment disorder (n=3).

Respondents with complete data were similar to those lost due to refusal and untracebility on the sociodemographic and treatment parameters.

Table 1. summarizes the pre-transplantation clinical characteristics of the whole sample, including the type of transplantation.

**Table 1:** Pretransplantation clinical characteristics of patients

**Category**	**Heart**	**Liver**	**Lung/heart and lung**
	**(n=62)**	**(n=43)**	**(n=21)**
**Indication for transplant**	Ischemic cardiomyopathy	Alcoholic liver disease	Emphysema/COPD
	34 (54.8%)	13 (30.2%)	6 (28.6%)
	Dilatative cardiomyopathy	Infectious hepatitis^a^	Pulmonary fibrosis
	28 (45.2%)	13 (30.2%)	7 (33.3%)
		Primary biliary cirrhosis	Pulmonary hypertension
		5 (11.6%)	3 (14.3%)
		Malignancy^b^	Other^d^
		6 (14.0%)	5 (23.8%)
		Miscellaneous^c^	
		6 (14.0%)	

^a^ Infectious hepatitis encompasses HBV-related and/or HCV-related liver diseases.

^b^ Malignancy includes hepatocellular carcinoma and other hepatic malignancies.

^c^ Miscellaneous comprises Wilson’s disease, Budd-Chiari syndrome, fulminant hepatic failure, and cryptogenic cirrhosis.

^d^ Other indications for lung transplant include lymphangioleiomyomatosis, cystic fibrosis, congenital cystic adenomatoid malformation of the lung.

Table 2. shows the sociodemographic characteristics of the whole sample, including the type of transplantation.

**Table 2:** Sociodemographic characteristics

**Category**	**All patients**	**Heart**	**Liver**	**Lung/heart and lung**	**p**
	**(n = 126)**	**(n = 62)**	**(n = 43)**	**(n = 21)**	
***Gender***					
**Male**	87 (69.0%)	51 (82.3%)	29 (67.4%)	7 (33.3%)	χ^2^=17.648; df=2;
**Female**	39 (31.0%)	11 (17.7%)	14 (32.6%)	14 (66.7%)	p<0.001^a^
***Age***					
**Mean (years)**	52.40	54.39	50.98	49.48	p=0.066^b^
**SD**	±11.64	±11.57	±11.72	±11.18	
***Marital status***					
**Single**	13 (10.3%)	6 (9.7%)	2 (4.7%)	5 (23.8%)	p=0.163^c^
**Married**	95 (75.4%)	50 (80.6%)	32 (74.4%)	13 (61.9%)	
**Widowed**	2 (1.6%)	1 (1.6%)	1 (2.3%)	-	
**Divorced**	16 (12.7%)	5 (8.1%)	8 (18.6%)	3 (14.3%)	
***Employment status***					
**Full-time**	26 (20.6%)	12 (19.4%)	13 (30.2%)	1 (4.8%)	p=0.011^c^
**Part-time/homeworker**	13 (10.3%)	5 (8.1%)	8 (18.6%)	-	
**Unemployment**	4 (3.2%)	3 (4.8%)	1 (2.3%)	-	
**Retired**	12 (9.5%)	9 (14.5%)	2 (4.7%)	1 (4.8%)	
**Disabled from work due to health-related resasons**	71 (56.3%)	33 (53.2%)	19 (44.2%)	19 (90.5%)	

*SD* Standard deviation.

^a^ χ^2^ tests.

^b^ Kruskal-Wallis one-way analysis of variance or ranks.

^C^ Fisher’s exact test.

### Posttraumatic Stress Symptoms (PTSS)

Before SOT, the psychometric observer-rating scale TERS together with an examination by experienced consultation-liaison psychiatrists was performed on all SOT candidates, and in no case were preoperative posttraumatic stress disorder (PTSD) or PTSS diagnosed.

After the surgical intervention 19 (15.1%) SOT recipients had clinical significant PTSS as measured on the PTSS-10 scale. The mean PTSS-10 score for the SOT recipients with PTSS was 44.6 (SD=6.9) points and for those without PTSS 18.9 (SD=6.8) points (Mann–Whitney-U=0.0; p<0.001). The average length of time since transplantation at the investigation point was similar in SOT recipients with and without clinically significant PTSS (SOT-recipients with PTSS 28.58 months; SOT-recipients without PTSS: 24.22 months; Mann–Whitney-U: 801.5, p=0.142).

### Preoperative variables with an influence on the risk of PTSS

Seven (43.8%) SOT recipients with PTSS and 12 (10.9%) patients without PTSS had a preoperative psychiatric diagnosis before SOT (χ^2^ =11. 765; df=1, p=0.001), suggesting an increased number of previous psychiatric illness in SOT recipients with PTSS. Frequent preoperative psychiatric diagnoses in the subgroup of SOT recipients with PTSS were adjustment disorder (n=5), alcohol dependence (n=2), histrionic personality disorder (n=2), dystymia (n=1), somatoform disorder (n=1)), obsessive compulsive disorder (n=1) and drug abuse (n=1).

SOT recipients with PTSS more frequently take benzodiazepines after the surgical intervention, but it needs to mentioned that even before the SOT, patients with postoperative PTSS had a higher level of benzodiazepines consumption (Fisher’s exact test, p=0.018). No differences between patients with PTSS and those without were found in the pre- and postoperative frequency of alcohol abuse (Fisher’s exact test, p=1.000).

Table 3. Pre- and postoperative frequency of benzodiazepine consumption and alcohol abuse.

**Table 3:** Pre- and postoperative frequency of benzodiazepine consumption and alcohol abuse

		**SOT recipients with post-operative PTSS**		**SOT recipients without post-operative PTSS**		
		**n**	**%**	**n**	**%**	**p**
**Alcohol**	**No alcohol abuse before and after SOT**	15	78.9	81	75.7	p=1.000^a^
	**Alcohol abuse before SOT**	3	15.8	21	19.6	
	**Current alcohol abuse after SOT**	1	5.3	5	4.7	
**Benzodiazepines**	**No benzo-diazepine consumption before and after SOT**	12	63.2	92	86	p=0.018^a^
	**Benzodiazepine consumption before SOT**	3	15.8	3	2.8	
	**Present benzo-diazepine consumption after SOT**	4	21.1	12	11.2%	

*PTSS* postoperative posttraumatic stress symptoms.

*SOT* solid-organ transplantation.

^a^ Fisher’s exact test.

Six liver transplant and one lung transplant recipients had a history of retransplantation and SOT recipients with a history of retransplantation suffered by trend more frequently from PTSS than those without (Fisher’s exact test, p=0.069).

Table 4. presents the history of retransplantations of SOT recipients with and without posttraumatic stress syndromes.

**Table 4:** The history of retransplantations of SOT recipients with and without posttraumatic stress syndromes

	**No retransplantation**		**Retransplantation**^**b**^		
	**n**	**%**	**N**	**%**	**p**
**SOT recipients with postoperative PTSS**	16	84.2	3	15.8	p=0.069^a^
			[One retransplantation: 2 (10.5%),		
			Two retransplantations: 1 (5.3)]		
**SOT recipients without post-operative PTSS**	103	96.3	4 (3.7%)	3.7	
			[One retransplantation: 4 (3.7%),		
			Two retransplantations: 0 (0%)]		

*PTSS* postoperative posttraumatic stress symptoms.

*SOT* solid-organ transplantation.

^a^ Fisher’s exact test.

^b^ Six liver transplant and one lung transplant recipients had a history of retransplantation.

No significant differences between SOT recipients with PTSS and those without were found on the following sociodemographic characteristics: gender (χ^2^ =0.363; df=1, p=0.594), marital status (Fisher’s exact test, p=0.555), years in education and/or vocational training (Mann–Whitney-U=988.5; p=0.846) and employment status (Fisher’s exact test, p=0.092).

SOT recipients with PTSS were younger than those without (mean age of transplantation recipients with PTSS 48.8 years [SD=10.1]; mean age of transplantation recipients without PTSS 53.0 years [SD=11.8]; Mann–Whitney-U=719.0; p=0.042).

### Posttraumatic stress symptoms and type of transplantation

Patients with liver transplantation displayed a higher level of PTSS after the surgical intervention than patients with heart or lung transplantation (Fisher’s exact test, p=0.003).

Table 5. illustrates PTSS according to type of transplantation.

**Table 5:** Posttraumatic stress symptomatology according to type of transplantation

	**Liver transplantation**		**Heart transplantation**		**Lung transplantation**		**p**
	**n**	**%**	**n**	**%**	**n**	**%**	
**SOT recipients with post-operative PTSS**	13	30.2%	4	6.5%	2	9.5%	p=0.003^a^
**SOT recipients without postoperative PTSS**	30	69.8%	58	93.5%	19	90.5%	

*PTSS* postoperative posttraumatic stress symptoms.

^a^ Fisher’s exact test.

### Posttraumatic stress symptoms and postoperative medical complications

The occurrence (χ^2^ =1.178; df=2, p=0.585) and the number (Fisher’s exact test; p=0.933) of postoperative medical complication (e.g. bleeding, infections, cardio-vascular) did not significantly differ in the liver transplantation recipients in our study from that in heart and lung transplantation recipients with the exception that liver and lung transplantation recipients had significantly more frequently acute rejections (χ^2^ =9.401; df=2, p=0.009). Furthermore, the incidence of posttraumatic stress symptoms after transplantation was independent of postoperative medical complications (χ^2^ =0.676; df=1, p=0.462).

### Health-related Quality of Life (HRQOL)

In comparison with those SOT recipients without PTSS, transplantation recipients suffering from PTSS displayed significant impairments in all HRQOL SF-36 domains: Physical Functioning, Role Physical, Pain, General Health, Vitality, Social Functioning, Role Emotional, and Mental Health.

Table 6. shows the HRQOL SF-36 domains according to PTSS.

**Table 6:** HRQOL SF-36 domains according to posttraumatic stress symptomatology

	**SOT recipients with postoperative PTSS**		**SOT recipients without postoperative PTSS**			
	**(n=19)**		**(n=107)**			
	**Mean**	**SD**	**Mean**	**SD**	**Mann–Whitney-U**	**p**
**Physical functioning**	40.3	±26.0	68.8	±26.0	415.0	p<0.001^a^
**Role physical**	25.0	±39.1	57.7	±43.2	558.0	p<0.001^a^
**Pain**	34.8	±23.9	69.1	±29.4	382.5	p<0.001^a^
**General health**	31.2	±17.8	61.2	±19.2	253.0	p<0.001^a^
**Vitality**	31.3	±17.9	57.8	±19.52	328.0	p<0.001^a^
**Social functioning**	53.3	±23.5	85.0	±18.1	290.5	p<0.001^a^
**Role emotional**	35.1	±45.1	81.7	±34.4	440.5	p<0.001^a^
**Mental health**	41.7	±18.4	78.7	±13.5	99.0	p<0.001^a^

*PTSS* posttraumatic stress symptoms.

*SOT* solid-organ transplantation.

^a^ Mann–Whitney-U-test; alpha adjustment (Bonferroni): p<0.006.

As mentioned above, SOT recipients without PTSS have significant lower impairments in all HRQOL domains than those with PTSS. However, compared with healthy controls matched by age and gender, even SOT recipients without PTSS have impairments in some HRQOL SF-36 domains: Physical Functioning (Wilcoxon-Z=−7.434, p<0.001), Role Physical

(Wilcoxon-Z=−6.452, p<0.001), Pain (Wilcoxon-Z=−5.075, p<0.001), General Health (Wilcoxon-Z=−4.434, p<0.001), Vitality (Wilcoxon-Z=−4.112, p<0.001), Social Functioning (Wilcoxon-Z=−4.679, p<0.001), and Role Emotional (Wilcoxon-Z=−3.225, p=0.001).

### Life satisfaction

In comparison with those SOT recipients without PTSS, transplantation recipients showing PTSS displayed significant impairments in the life satisfaction questionnaire (FLZ) global score and in the life satisfaction domains health and character/own skills.

Table 7. shows the life satisfaction domains (FLZ) according to PTSS.

**Table 7:** Life satisfaction domains (FLZ) according to PTSS

	**SOT recipients with PTSS**		**SOT recipients without PTSS**			
	**Mean**	**SD**	**Mean**	**SD**	**Mann–Whitney-U**	**p**
**FLZ Global Score**	241.0	±29.8	274.4	±29.2	228.0	p=0.002^a^
**Health**	23.2	±9.7	38.4	±7.0	174.0	p<0.001^a^
**Occupation/work**	28.6	±16.4	39.0	±6.8	407.0	p=0.028^a^
**Income/financial security**	27.9	±13.0	37.0	±8.3	467.0	p=0.013^a^
**Leisure time/hobbies**	34.1	±11.2	40.1	±6.8	548.0	p=0.036^a^
**Partner relationship**	37.3	±12.4	43.2	±7.1	395.0	p=0.039^a^
**Family life/children**	38.2	±13.4	40.7	±7.7	407.5	p=0.839^a^
**Character/own skills**	34.8	±7.4	40.8	±3.8	401.5	p=0.001^a^
**Sexuality**	33.1	±11.5	35.2	±8.5	518.5	p=0.538^a^
**Friends/acquaintances**	34.3	±8.3	39.0	±5.3	544.5	p=0.029^a^
**Housing/living conditions**	39.4	±5.5	42.6	±4.7	529.0	p=0.021^a^

*PTSS* posttraumatic stress symptoms.

*SOT* solid-organ transplantation.

^a^ Mann–Whitney-U-test; alpha adjustment (Bonferroni): p<0.005.

### Multivariate analyses

In the univariat analyses described so far the following risk factors for the display of PTSS after SOT were identified: 1.) preexisting psychiatric morbidity, 2.) history of retransplantation (with the limitation that in our sample only patients with liver or lung transplantations had retransplantations in the past), 3.) chronic benzodiazepine consumption, 4.) age, and 5.) type of transplantation. In the second step a multivariate analysis in the form of a multiple-regression model was used to examine the levels of influence of the identified risk factors of PTSS. The following predictors were selected in the prediction of PTSS-10 values: preexisting psychiatric morbidity, history of retransplantation, benzodiazepine consumption before transplantation, age, and type of transplantation. Added to the list of predictors were medical complications, survival time since transplantation and gender. This model yields a R^2^ (adj.) =0.145, F=3.125, p=0.002 and shows that significant predictors of PTSS post-SOT are preexisting psychiatric morbidity and history of retransplantation.

In a third step, an additional multiple regression model was calculated that included the PTSS-associated dimensions in HRQOL (SF-36) and life satisfaction (FLZ) identified in the univariat analyses as well as benzodiazepine consumption after SOT. This model yields R^2^ (adj.) =0.562, F=37.229, p<0.001 and shows the statistically significant associative relationship between impaired HRQOL (SF-36 - physical health subscales: p<0.001; SF-36 mental health subscales p<0.001) and life satisfaction (FLZ - occupation, work: p=0.004) and PTSS-10 values.

Table 8. Multiple regression analyses.

**Table 8:** Multiple regression analyses

**Model**	**R**	**R**^**2**^	**R**^**2 **^**(adj.)**	**F**	**p**
**Model 1: risk factors for PTSS**	0.461	0.213	0.145	3.125	0.002
**Model 2: PTSS associated dimensions in HRQOL and life satisfaction**	0.760	0.577	0.562	37.229	<0.001
**Model**	**Predictor**	**B**	**Beta**	**p**	
**Model 1:**	Age	0.069	0.074	0.437	
	Gender	2.316	0.099	0.320	
	Preexisting psychiatric morbidity	6.505	3.067	**0.036**	
	History of retransplantation	11.586	4.836	**0.018**	
	Benzodiazepine consumption before transplantation	3.574	0.674	0.502	
	Type of transplantation - liver	2.281	0.100	0.326	
	Type of transplantation - lung	−1.373	−0.051	0.623	
	Medical complications	2.640	0.125	0.179	
	Survival time since transplantation	0.129	0.146	0.115	
**Model 2:**	SF-36: physical health	−0.283	−0.3.331	**<0.001**	
	SF-36: mental health	−0.538	−0.531	**<0.001**	
	FLZ – occupation/work	−0.226	−0.202	**0.004**	
	Benzodiazepine consumption after transplantation	2.255	0.070	0.279	

## Discussion

Although the standardizations of surgical procedures and improvements in intensive care treatment, patient selection, tissue matching and organ preservation have advanced the success of SOTs, PTSS remain a frequent finding in post-SOT patients, and until now only few empirical data exist about the prevalence of PTSS among patients undergoing SOTs [30,31]. PTSS after SOT can cause compliance problems and decreases long-term survival [32]. In our study 19 (15.1%) SOT recipients displayed PTSS. This rate is consistent with previous research. For example, in an orthotopic liver transplantation study by Rothenhäusler et al. [24], 2.7% suffered from full PTSD and 16% from partial PTSD 3.8 years after transplantation. In another study by Favaro et al. [33], the estimated frequency of transplantation-related PTSS after heart transplantation was 12%. PTSD in SOT recipients may indicate a failure of the patient to come to terms with the transplant experience and Dew et al. reported that the risk of mortality increases in patients who met criteria for postraumatic stress disorder after heart transplantation [16,34]. Little is known about predictors and risk factors of posttraumatic stress symptomatology after SOT. Favaro et al. [33] describe a preoperative history of depressive episodes and perceived social support as risk factors of postoperative posttraumatic stress symptomatology.

Dew et al. [16,34-36] evaluated in a longitudinal study risk factors for posttraumatic stress disorder related to transplantation during the first year after heart transplantation. The following pre-transplant and perioperative risk factors increased recipients’ risk of any psychiatric disorder: pre-transplant psychiatric history, poor social support, the use of avoidance coping strategies for managing health problems, and low self esteem early posttransplant. Regarding PTSD, the waiting period for transplant appeared to be the most traumatic experience for many SOT recipients.

In analysing the data of our study we identified the following additional preoperative risk factors for developing postoperative PTSS symptomatology after SOT: 1.) a history of liver or lung retransplantation, 2.) preoperative chronic benzodiazepine consumption, 3.) age, and 4.) type of transplantation. Dew et al. [34] previously have reported, that a pre-existing psychiatric morbidity is a preoperative risk factor for developing postoperative PTSS symptomatology. Our study also confirms this finding. In comparison with heart or lung transplantation recipients, the subgroup of liver transplantation recipients observed in this study had greater preoperative psychiatric comorbidity and a higher risk for developing PTSS after the transplantation.

A multiple regression analysis identified a preexisting psychiatric morbidity and a history of retransplantation as significant predictors of PTSS in SOT recipients. The occurrence of postoperative medical complications (e.g. bleeding, infections, cardio-vascular, rejection) did not significantly differ in SOT patients with postoperative PTSS from those without PTSS.

HRQOL and life satisfaction are an important measure of outcome after SOT [11,30], and in recent years, several studies have demonstrated that SOT has beneficial effects on the health-related quality of life for the majority of patients, both in intermediate and in long-term outcomes [24]. In addition, Goetzmann et al. [37] showed in a prospective study among SOT recipients that an organ transplant significantly improved posttransplant life satisfaction [37]. Dew et al. [38] found, that emotional well-being was stable or improved in the first 12 months after heart transplantation and in addition the physical functional status on standard functional measures of sleep, body care, mobility and ambulation improved in the same period significant.

In this present study even SOT recipients without PTSS displayed more impairments in some HRQOL domains than healthy controls matched by age and gender. It is recognised that PTSS impairs HRQOL and life satisfaction in people with overall good health. Therefore a major aim of the present study was to explore the associations between impairments in HRQOL and life satisfaction of transplant patients suffering from concomitant PTSS. The SOT recipients with PTSS examined in this study had a lower HRQOL compared with patients without PTSS and impairments in the following HRQOL SF-36 domains were found: Physical Functioning, Role Physical, Pain, and General Health in the physical health SF-36 subscales, and Vitality, Social Functioning, Role Emotional and Mental Health in the mental health SF-36 subscales.

Furthermore the life satisfaction domains (FLZ) Health and Character/Own Skills were affected. In addition a multivariate analysis showed the statistically significant associative relationship between impaired HRQOL (SF-36 physical health subscales, SF-36 mental health subscales), life satisfaction (occupation, work) and PTSS-10 values.

In conclusion our results suggest that PTSS are strongly associated with massive additional impairments of HRQOL and life satisfaction in SOT recipients.

**Figure 1:**
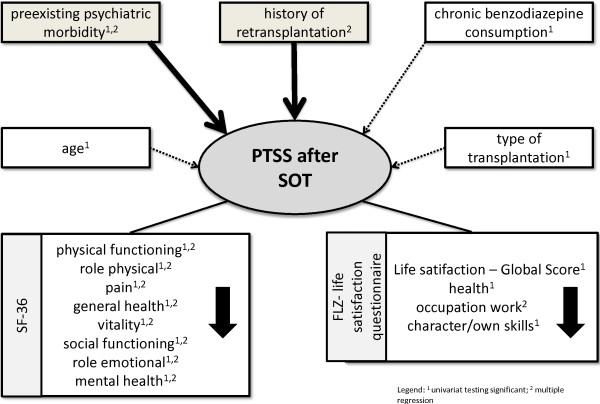
**Posttraumatic stress symptoms (PTSS) after solid-organ transplantation (SOT) - risk factors and the impact on health-related quality of life and life satisfaction. **^1^ univariat testing significant; ^2^ multiple regression.

Figure 1. PTSS after solid-organ transplantation (SOT) - risk factors and the impact on health-related quality of life and life satisfaction.

### Limitations

Finally, there are several limitations to this study that deserve mention. The format of the study is a retrospective follow-up, and the absence of a prospective randomized trial makes it difficult to definitively conclude that the HRQOL associations observed are causal in nature.

The response rate is similar to that reached in other comparable studies (e.g. Rothenhäusler et al. [24]). As the eligible SOT recipients were asked in a letter, not in a face-to-face follow-up check, to participate in this study reasons for refusal could not be collected in all patients. A possible skewing of the study results due to the response rate of 59% cannot definitely be ruled out. The PTSS-10 is not sufficient for the diagnosis of posttraumatic stress disorder. Therefore elevated PTSS-10 scores were only classified as posttraumatic stress symptoms. Pre-transplant psychosocial assessments might be distorted due to the point, that SOT candidates are concerned that any information that they impart could influence the transplant team’s decisions about the patient’s candidacy. Furthermore, the study population consists of a mixed group of patients with different organ entities, in whom transplantation has been conducted and the small group size of the organ-subgroups is a limitation to differ between the different subgroups. However, in regard to posttraumatic stress symptoms common data interpretation is possible due to the homogeneously trauma criterion definition as SOT followed by ICU treatment. Finally, preoperative psychiatric diagnoses based on the TERS and an examination by consultation-liaison psychiatrists. Additional structured interviews have not been conducted to secure the diagnoses.

## Conclusions

SOT recipients may face major transplantation- and treatment-related PTSS and impairments to their HRQOL and life satisfaction. Much speaks in favour of searches for preoperative risk factors that might help to predict postoperative PTSS. Advance warning of the threats of this condition could potentially reduce significant morbidity, mortality, and compliance problems. Further, PTSS are highly associated with impairments to patients’ overall quality of life and life satisfaction. In conclusion we advise that as part of routine clinical care, an early and comprehensive bio- and psychosocial diagnosis and therapeutic treatment of SOT patients to be carried out, so that their posttraumatic stress symptomatology can be treated rapidly and their quality of life and life satisfaction can be improved.

## Abbreviations

FLZ: Fragebogen zur Lebenszufriedenheit, life satisfaction questionnaire; HLT: Heart and lung transplantation; HRQOL: Health-related quality of life; HT: Heart transplantation; ICU: Intensive care unit; LT: Lung transplantation; OLT: Orthotopic liver transplantation; PTSD: Posttraumatic stress disorder; PTSS: Posttraumatic stress symptoms; SD: Standard deviation; SF-36: Medical Outcome Study Form SF-36; SOT: Solid-organ transplantation; TERS: Transplant evaluation rating scale.

## Competing interests

The authors declare that they have no competing interests.

## Authors’ contributions

AB: participated in data analysis; participated in the writing of the paper. TK: participated in research design; participated in the performance of the research. HBR: participated in research design; participated in the performance of the research, participated in the writing of the paper. All authors read and approved the final manuscript.
